# Silibinin sensitizes chemo-resistant breast cancer cells to chemotherapy

**DOI:** 10.1080/13880209.2016.1270972

**Published:** 2016-12-27

**Authors:** Ommoleila Molavi, Farzaneh Narimani, Farshid Asiaee, Simin Sharifi, Vahideh Tarhriz, Ali Shayanfar, Mohammadsaied Hejazi, Raymond Lai

**Affiliations:** a Biotechnology Research Center, Tabriz University of Medical Sciences, Tabriz, Iran;; b Faculty of Pharmacy, Tabriz University of Medical Sciences, Tabriz, Iran;; c Department of Laboratory Medicine and Pathology, Faculty of Medicine and Dentistry, University of Alberta, Edmonton, Alberta, Canada

**Keywords:** Resistance, doxorubicin, paclitaxel, apoptosis, STAT3 pathway

## Abstract

**Context:** Multiple drug resistance is the major obstacle to conventional chemotherapy. Silibinin, a nontoxic naturally occurring compound, has anticancer activity and can increase the cytotoxic effects of chemotherapy in various cancer models.

**Objective:** To evaluate the effects of silibinin on enhancing the sensitivity of chemo-resistant human breast cell lines to doxorubicin (DOX) and paclitaxel (PAC).

**Materials and methods:** The cells were treated with silibinin (at 50 to 600 μM concentrations) and/or chemo drugs for 24 and 48 h, then cell viability and changes in oncogenic proteins were determined by MTT assay and Western blotting/RT-PCR, respectively. Flow cytometry was used to study apoptosis in the cells receiving different treatments. The antitumorigenic effects of silibinin (at 200 to 400 μM concentration) were evaluated by mammosphere assay.

**Results:** Silibinin exerted significant growth inhibitory effects with IC_50_ ranging from 200 to 570 μM in different cell lines. Treatment of DOX-resistant MDA-MB-435 cells with silibinin at 200 μM reduced DOX IC_50_ from 71 to 10 μg/mL and significantly suppressed the key oncogenic pathways including STAT3, AKT, and ERK in these cells. Interestingly treatment of DOX-resistant MDA-MB-435 cells with silibinin at 400 μM concentration for 48 h induced a 50% decrease in the numbers of colonies as compared with DMSO-treated cells. Treatment of PAC-resistant MCF-7 cells with silibinin at 400 μM concentration generated synergistic effects when it was used in combination with PAC at 250 nM concentration (CI = 0.81).

**Conclusion:** Silibinin sensitizes chemo-resistant cells to chemotherapeutic agents and can be useful in treating breast cancers.

## Introduction

Breast cancer is the most common type of cancer in women, with more than one million newly reported cases per year (Donepudi et al. 2014). The most commonly used strategy for treatment of breast malignancies is conventional chemotherapy. The main reason behind the failure of chemotherapy in breast cancer is the development of multidrug resistance (MDR). MDR refers to the acquired or inherent resistance of cancer cells to multiple drugs by different mechanisms including the increased efflux of chemotherapeutic agent from cancer cells, blocked apoptosis, decreased drug influx, altered intracellular drug localization and altered cell cycle regulation (Baguley 2010; Tan et al. 2014). Over the last two decades, extensive efforts have been devoted to identify the molecular targets responsible for MDR and development of effective inhibitors for such targets. Many MDR reversing agents have been developed from medicinal plants, which are rich sources of phytotherapeutic agents (Syed & Coumar 2016).

Silibinin is a flavonoid antioxidant from milk thistle [*Silybum marianum* (L.) Gaertn (Asteraceae)], which has been used for the treatment of liver diseases for many years (Ferenci et al. 1989). An increased number of recent *in vitro* and *in vivo* studies have shown the effects of silibinin on growth inhibition, cell cycle arrests and induction of apoptosis in several types of cancer including lung, prostate, breast and lymphomas (Zhang et al. 2012; Ting et al. 2013; Pirouzpanah et al. 2015; Molavi et al. 2016). Previous studies have also reported a synergistic anti-proliferative effect of silibinin when given in combination with commonly used chemotherapeutic agent such as doxorubicin (DOX) and paclitaxel (PAC) (Raina & Agarwal 2007). Nevertheless, the effects of silibinin on restoring the sensitivity of chemo-resistant cancers have not been fully investigated. In the present study, we evaluated the effects of silibinin on enhancing the sensitivity of chemo-resistant MCF-7 and MDA-MB-435 breast cancer cell lines to two widely used chemotherapeutic agents, DOX and PAC. Here, we also studied the effects of silibinin on STAT3, an oncogenic pathway, in DOX-resistant MDA-MB-435 cells which contain constitutively active STAT3. Several previously published papers have shown that constitutive activation of STAT3 plays an important role in the development of MDR in cancer cells. While there are a few reports on the inhibitory effects of silibinin on STAT3 pathway in cancer cells, to our knowledge the effects of silibinin on STAT3 and MDR in drug-resistant cancer cells harbouring hyperactive STAT3 have not been reported before.

## Materials and methods

### Materials

DOX (doxorubicin hydrochloride 98%) was obtained from Ontario Chemicals Inc. (Ontario, Canada). RPMI-1640 culture media and FBS (foetal bovine serum) were purchased from Sigma (Sigma-Aldrich, St. Louis, MO). MTT reagent and silibinin were obtained from Sigma. PAC was from Actavis (Nerviano, Italy) and annexin V/Propidium Iodide (PI) kit was from BD Biosciences (Mississauga, ON). All other chemicals were of analytical grade.

### Cell lines

The wild-type human MDA-MB-435 cancer cell line (MDA-MB-435/WT) was received as a gift from the laboratory of Dr R. Clarke (Georgetown University, USA). The DOX-resistant phenotype of MDA-MB-435 (MDA-MB-435/DOX) was provided as a gift by the laboratory of Dr H. Uludag (University of Alberta, Canada). This cell line was developed through culture of MDA-MB-435/WT cells in the presence of low DOX concentrations as reported before (Falamarzian et al. 2014). MDA-MB-435/DOX cells were cultured in the presence of 2 μg/mL of DOX in culture media at all times. The wild type human breast adenocarcinoma cell line, MCF-7, (MCF-7/WT) was purchased from Pasteur Institute of Iran (Tehran, Iran). The paclitaxel-resistant MCF-7 cell line (MCF-7/PAC) was developed through culture of MCF-7/WT cells in the presence of low PAC concentrations as reported previously (Sharifi et al. 2014). MCF-7/PAC cells were cultured at 64 nM concentration of PAC at all times. All the cell lines were cultured in RPMI 1640 medium supplemented with 100 U/mL penicillin, 100 μg/mL streptomycin, and 10% FBA in a humidified atmosphere containing 5% CO_2_ at 37 °C.

### Cytotoxicity assay

The *in vitro* cytotoxicity was evaluated by 3-(4,5-dimethylthiazol-2-yl)-2,5-diphenyltetrazolium bromide (MTT) assay. Cells were seeded at a density of 0.5 × 10^4^ cells per well in 200 μL growth medium in 96-well plates and grown overnight. The cells were then challenged with different concentrations of either compounds (silibinin, DOX or PAC) or combination of silibinin with either one of chemotherapeutic agents (DOX or PAC). Untreated cells and DMSO-treated cells were used as control cells. After incubation for 24 and 48 h, the media was replaced with fresh culture media containing MTT solution (0.5 mg/mL), and the cells were incubated for an additional 4 h at 37 °C. Then, the medium was removed and DMSO was added to dissolve the formazan crystal formed by living cells. The absorbance was measured by a microplate reader (PowerWave340™, BioTek Instruments, Inc. USA) at dual wavelengths of 570 and 650 nm. Cell viability was calculated by comparing the absorbance in the cells treated with drug with that in the control cells which were only added with DMSO. The read-outs are the percentages of viable cells in drug-treated cells relative to those of DMSO-treated cells (i.e. negative controls). IC_50_ value (inhibitory concentrations required to reduce the cell viability by 50%) of drugs was estimated from a plot of the percentage of viable cells versus drug concentration. Using data obtained from MTT assays and CompuSyn software, the dose-effect curves for single agents and their combinations were generated. The combination index (CI) values for each dose and the corresponding effect level, referred to as the fraction affected (f_a_; the fraction of cells inhibited after the drug exposure, e.g., 0.5 when cell growth is inhibited by 50%), were calculated. The CI values at f_a _= 0.5 have been reported for the combinational therapies in the present research work. The combination index offers a quantitative definition for an additive effect (CI = 1), synergism (CI < 1), and antagonism (CI > 1) in drug combinations (Chou 2010).

### Cell death and cell growth assays

To examine the effects of silibinin alone or in combination with chemo drugs on the induction of cell death in the breast cancer cell lines, the cells were counted after 48 h of incubation with the drugs and then equal number of cells from each group were collected and stained with Annexin/PI according to the manufacturer’s instructions. The samples were then immediately analyzed on a Becton Dickinson FACSCalibur flow cytometer (Franklin Lake, NJ, and USA). To assess the status of the cell cycle in breast cell lines, the cells were stained with PI and cell-cycle analysis was performed by flow cytometry as reported previously (Wu et al. 2015). In brief, the cells after different treatments for 48 h were fixed with ice-cold 70% ethanol then subjected to RNase treatment and PI staining. After 30 min incubation with PI at room temperature, the cells were washed and immediately analyzed on a Becton Dickinson (Franklin Lakes, NJ, USA) FACSCalibur flow cytometer. Data acquisition was gated to exclude cell doublets, and the cell-cycle phase distribution was determined using the CellQuest program (20 000 events were counted). The experiments were performed in triplicate and repeated on two separately initiated cultures.

### Western blotting

Western blot was performed as described previously (Molavi et al. 2013). All the antibodies were diluted in 5% bovine serum albumin (BSA) in Tris buffered saline and 0.1% Tween-20 (TBST); all of the first and secondary antibodies were purchased from Cell Signaling Technologies (Danvers, MA). The expression of β-actin served as the loading control for all western blot studies.

### RT-PCR

RNA extraction of the cells was done by a RNA extraction kit, RNX-Plus (CinnaGen, Tehran, Iran) according to the protocol. Quality of extracted RNA was qualified by agarose gel electrophoresis. Concentration of the extracted RNA was determined by optical density measurement (A260/A280 ratio) with NanoDrop 1000 Spectrophotometer (Wilmington, DE). RNA was converted to cDNA using REVERTA-L RT reagents kit (AmpliSens, Moscow, Russia) and then the reaction tubes were incubated at 42 °C for 60 min. Real time PCR was carried out using the SYBR Green-based PCR Master Mix and analyzed on a Corbett 6000 Rotor-Gene thermocycler (Corbett Research). The following primer sets were used: BAX: 5′-GATGCGTCCACCAAGAAG-3′ (F) and 5′-AGTTGAAGTTGCCGTCAG-3′ (R); Bcl2: 5′-CATCAGGAAGGCTAGAGTTACC-3′ (F) and 5′-CAGACATTCGGAGACCACAC-3′ (R); Survivin: 5′-CAGATTTGAATCGCGGGACCC-3´ (F) and 5′-CCAAGTCTGGCTCGTTCTCAG-3′ (R). Total volume of amplification reactions was 25 μL and each well was included 12.5 μL of SYBR Green PCR Master Mix, 1 μL of cDNA, 70-100 nM of both forward and reverse special primers. The PCR thermal cycling steps were included 10 min at 95 °C, 40 cycles of 20 s at 95 °C for denaturation step, 25 s at annealing temperature, and 20 s at 72 °C for the extension, respectively. Final 10 min incubation at 72 °C was carried out to completion of amplicons. The relative expression levels of BAX, Bcl2 and surviving were calculated by normalizing the cycle threshold values of these proteins with those of β-actin.

### Mammosphere assay

Mammospheres were cultured as previously described (Wu et al. 2012). Briefly MDA-MB435/WT and MDA-MB435/DOX cells were treated with silibinin at the concentration of 250 and 400 μM, respectively. After 48 h incubation, the cells detached by trypsin, passed through a 40 μm cell strainer (BD, Franklin Lakes, NJ) and seeded into ultra-low adherent plates (Corning) in MammoCult media (StemCell Technologies, Vancouver, BC, Canada). The colonies were counted after 7 days.

### Statistical analysis

Data represent the mean and standard deviation in each experiment which was repeated at least three times. Difference between the mean values were analyzed by two-sided unpaired Student’s *t* test or One Way ANVOA, followed by Tukey's *post hoc* tests, *p* < 0.05 is denoted by * and *p* < 0.01 is denoted by ** in the figures.

## Results

### Silibinin inhibits cell growth and enhances the anticancer effects of DOX in MDA-MB-435/WT cells

**Figure 1.**
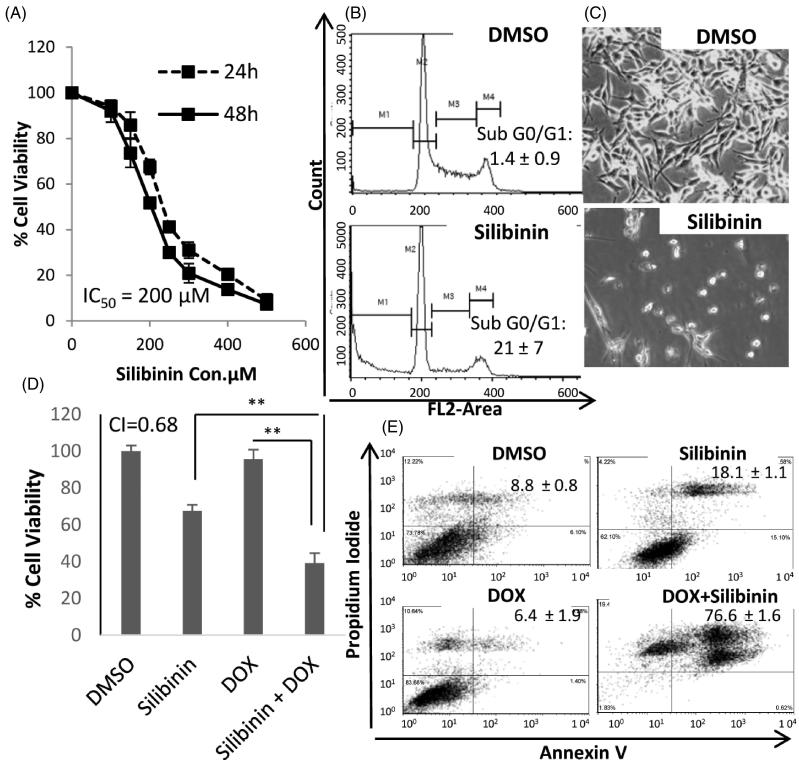
Growth inhibitory and chemo-sensitizing effects of silibinin in MDA-MB 435/WT cell line. (A) The dose-response cure for the growth inhibitory effects of silibinin assessed by MTT. (B) Cell cycle analysis by PI staining and flow cytometry. (C) Morphological alterations in MDA-MB-435/WT cells at 48 h post incubation with silibinin at concentration of 200 μM (40x magnification). (D) The synergistic growth inhibitory effects of silibinin and DOX measured by MTT. (E) Analysis of apoptosis by AnnexinV/PI assay.

**Figure 2.**
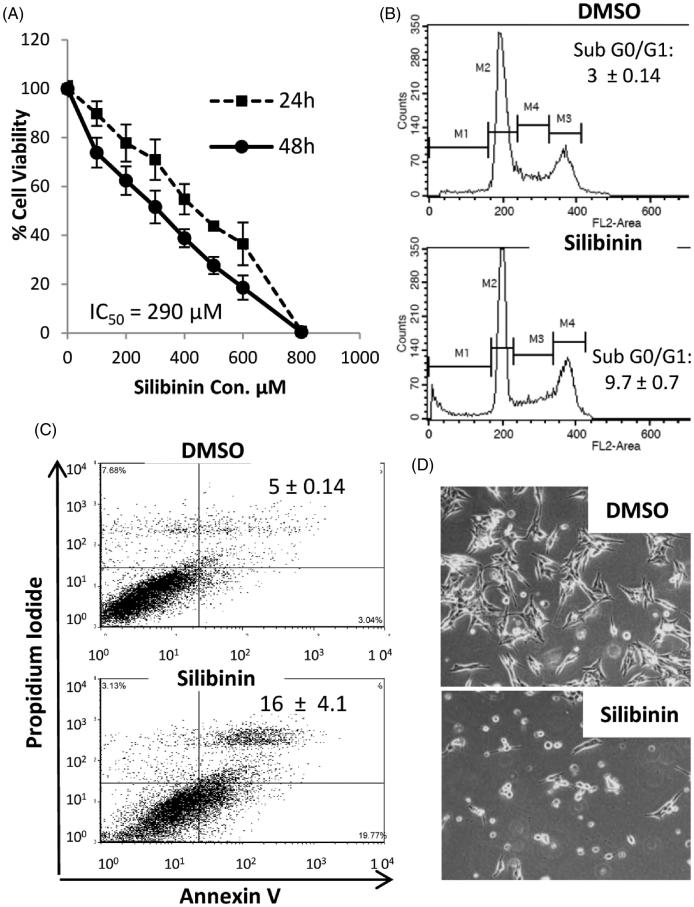
Anticancer effects of silibinin in MDA-MB-435/DOX cells. (A) The dose-response cure for the growth inhibitory effects of silibinin assessed by MTT. (B) Cell cycle analysis by PI staining and flow cytometry. (C) Analysis of apoptosis by AnnexinV/PI assay. (D) Morphological alterations in MDA-MB-435/DOX cells at 48 h post incubation with silibinin at concentration of 290 μM (40x magnification).

First, we assessed the anticancer effects of silibinin in MDA-MB-435/WT cells. The cells were treated with varying concentrations of silibinin, and the cell viability was quantified at 24 and 48 h as described in material and methods section. As shown in Figure 1(A), silibinin potently inhibited the growth of MDA-MB-435/WT cells with the IC_50_ being 200 μM at 48 h. Flow cytomeric analysis revealed that MDA-MB-435/WT cells treated with silibinin at 200 μM concentration for 48 h (i.e., the IC_50_ at 48) show a dramatic increase in the G0/G1 phase as compared with control cells treated with DMSO (Figure 2(B)). This increase in subG0/G1 is highly statistically significant, with the fraction of cells in subG0/G1 increased from 1.4% to 21%. Figure 1(C) shows dramatic changes in the morphology of MDA-MB-435/WT cells treated with 200 μM concentration of silibinin for 48 h.

Several studies have previously reported that the combination of silibinin with chemotherapeutic agents can result in synergistic anticancer effects (Tyagi et al. 2004; Raina & Agarwal 2007). To examine whether silibinin can increase the therapeutic efficacy of chemotherapy against MDA-MB-435/WT cells, the cells were treated with silibinin alone or in combination with DOX, which is a component of the current standard frontline regimen for breast cancer. As illustrated in Figure 1(D), treatment of MDA-MB-435/WT cell line with DOX (0.25 μg/mL) in combination with silibinin (200 μM) for 24 h resulted in a significant decrease in cell viability as compared with cells treated with either silibinin or DOX alone at the same concentration as combination therapy. It is worth noting that the IC_50_ of DOX in MDA-MB-435/WT cells is found to be 0.64 μg/mL, therefore, in the combinational treatment, we chose to use DOX at 0.25 μg/mL, the concentration at which DOX have a minimum effects on the loss of cell viability (about 7%). AnnexinV/PI analysis (Figure 1(E)) revealed a dramatic increase in the percentage of late apoptotic cells (Annexin V^+^/PI**^+^**) in MDA-MB-435/WT cells treated with combination of silibinin and DOX as compared with the cells treated with DOX only (76% vs. 6.4%). The percentage of the late apoptotic cells in the group treated with silbinin alone was 18% which was significantly lower than that in the group received silibinin in combination with DOX (76%) (*p* < 0.0001). Of note, we observed a small percentage of Annexin V^-^/PI**^+ ^**cells in the groups treated with DOX alone or in combination with silibinin, which indicates the occurrence of non-apoptotic (necrotic) mechanism of cell death. This observation is in line with previous studies showing that DOX induces necrotic death in cancer cells (Eom et al. 2005). Trypan blue assay and microscopic evaluation of MDA-MB-435/WT cells receiving different treatments at various time points showed dramatic changes in the morphology and viability of the cells received combinational therapy versus DMSO-treated cells or the cells that received monotherapy (Supplementary Figure 1).

### Silibinin suppresses the cell growth and induces apoptosis in MDA-MB-435/DOX cells

Next, we assessed the growth inhibitory effects of silibinin in MDA-MB-435/DOX cells. Silibinin inhibited the growth of MDA-MB-435/DOX cells in a dose-dependent manner (Figure 2(A)). The IC_50_ value for the cytotoxic effects of silibinin in MDA-MB-435/DOX cells was found to be 290 μM. Cell cycle analysis showed a significant increase in the percentage of cells in sub G0/G1 phase (Figure 2(B)) 48 h after treatment with silibinin at 290 μM concentration as compared with control cells. The percentage of cells in Sub G0/G1 increased from 3% in control group to 9.7% in the cells treated with silibinin. The cell cycle analysis results correlate with the data obtained from AnnexinV/PI apoptosis analysis showing approximately a three times increase (5% vs. 16%) in the percentage of late apoptotic cells (Annexin V^+^/PI**^+ ^**cells) in the group treated with silibinin as compared with control group (Figure 2(C)). As it is depicted in Figure 2(D), there is a dramatic change in the morphology of MDA-MB435/DOX cells after 48 h treatment with silibinin at 290 μM concentration.

### Silibinin sensitizes MDA-MB-435/DOX cells to chemotherapy

**Figure 3.**
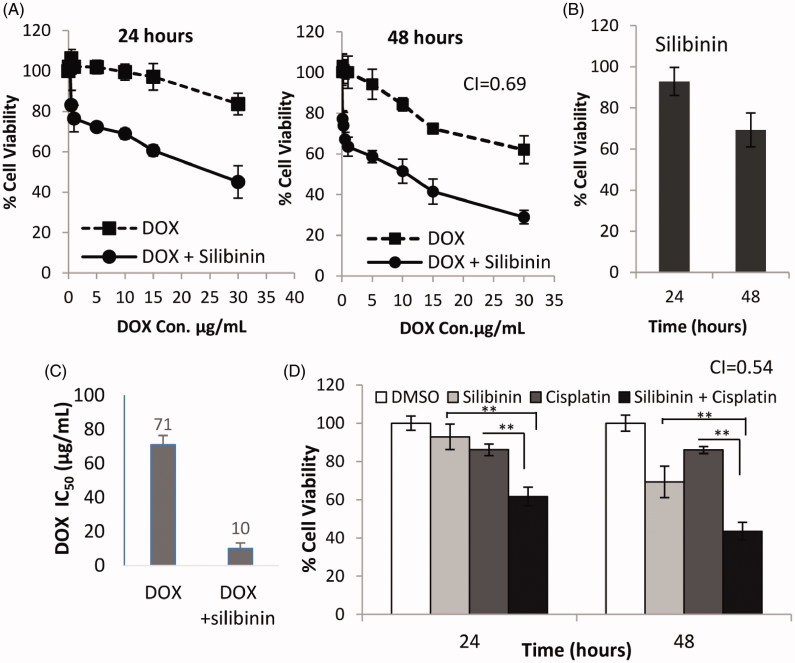
Synergistic anticancer effects of silibinin and DOX in MDA-MB-435/DOX cells. (A) Growth inhibitory effects of DOX in combination with 200 μM concentration of silibinin measured by MTT. (B) Growth inhibitory effects of silibinin alone at 200 μM concentration. (C) The effects of silibinin on IC_50_ of DOX in MDA-MB-435/DOX cells. (D) The growth inhibitory effects of cisplatin alone or in combination with silibinin at 200 μM concentration in MDA-MB-435/DOX cells.

As mentioned above, silibinin has been shown to enhance the cytotoxic effects of many chemotherapeutic drugs in various types of cancer. Thus we hypothesized that silibinin may sensitize MDA-MB-435/DOX cells to the killing effects of chemotherapeutic agents (i.e., DOX and cisplatin). In support of our hypothesis, we found that treatment of MDA-MB-435/DOX with combination of silibinin at 200 μM and DOX at various concentrations (5–40 μg/mL) significantly reduces the cell viability at all DOX concentrations as compared with the cells treated with DOX alone (Figure 3(A)). Figure 3(B) shows growth inhibitory effects of silibinin alone at 200 μM concentration in MDA-MB- 435/DOX cells. As it is summarized in Figure 3(C) silibinin (200 μM) reduces the IC_50_ of DOX from 71 to 10 μg/mL in MDA-MB-435/DOX cells. CI for the anticancer effects of silibinin in combination with DOX was found to be 0.69 indicating a synergism between these two drugs. Correlating with this data, we found that silibinin significantly enhances the cytotoxic effects of cisplatin in MDA-MB-435/DOX cells. As it is shown in Figure 3(D), the cell viability in the cells treated with cisplatin at 1 μg/mL concentration in combination with silibinin at 200 μM for 48 h was about 43% which was significantly lower than that in the cells treated with cisplatin (85%) or silibinin (70%) alone at the same concentrations as combination therapy for 48 h. The growth inhibitory effects of silibinin and cisplatin combination therapy was found to be a synergistic effect (CI = 0.54).

### Silibinin suppresses STAT3 in MDA-MB-435/WT and MDA-MB435/DOX cells

**Figure 4.**
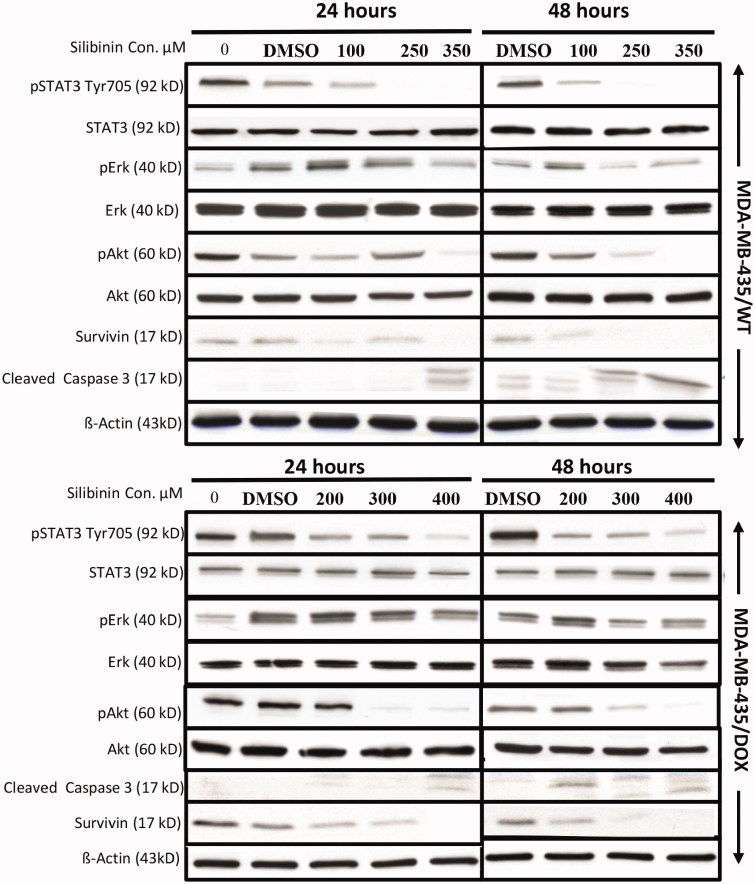
Suppressive effects of silibinin on key oncogenic pathways in MDA-MB-435/WT and MDA-MB-435/DOX cell lines. Western blotting was used to measure the level of oncogenic proteins in the cells treated with an increasing concentration of silibinin. These results are representative of three independent experiments.

It has been previously reported that silibinin suppresses the activation of STAT3 leading to the inhibition of cell growth and induction of apoptosis in a number of cancer cell lines *in vitro* (Bosch-Barrera & Menendez 2015). STAT3 is found constitutively active in many types of human malignancies and play a key role in cancer progression (Al Zaid Siddiquee & Turkson 2008; Wang et al. 2012). Recent studies have also shown that constitutive activation of STAT3 can mediate resistance to chemotherapy (Real et al. 2002; Tan et al. 2014). MDA-MB435 breast cancer cell line has constitutively active STAT3 which has been shown to be important for their growth and resistance to chemotherapy (Falamarzian et al. 2014). Therefore, we investigated whether the suppression of STAT3 by silibinin results in the inhibition of cell growth in these cells and sensitize them to chemotherapy. As it is shown in Figure 4, treatment of both MDA-MB-435/WT and MDA-MB-435/DOX with silibinin at increasing concentration resulted in suppression of STAT3 (indicated by p-STAT3 level) in a dose-dependent manner. To further analyze the mechanisms of silibinin anticancer activity in MDA-MB435 cells, we evaluated the inhibitory effects of this compound on two other oncogenic pathways, AKT and ERK, both of which play an important role in cancer cells growth and their resistance to chemotherapy (Dhillon et al. 2007; Steelman et al. 2011; Mundi et al. 2016). As depicted in Figure 4, silibinin significantly suppressed ERK and AKT pathways in both MDA-MB-435/WT and MDA-MB-435/DOX cell lines. The induction of apoptosis induced by silibinin was also supported by an increase in the level of cleaved caspase 3 (ClC_3_) following silibinin treatment in MDA-MB-435/WT and MDA-MB-435/DOX cell lines.

### Silibinin suppresses the tumorigenic properties of MDA-MB435 cells

**Figure 5.**
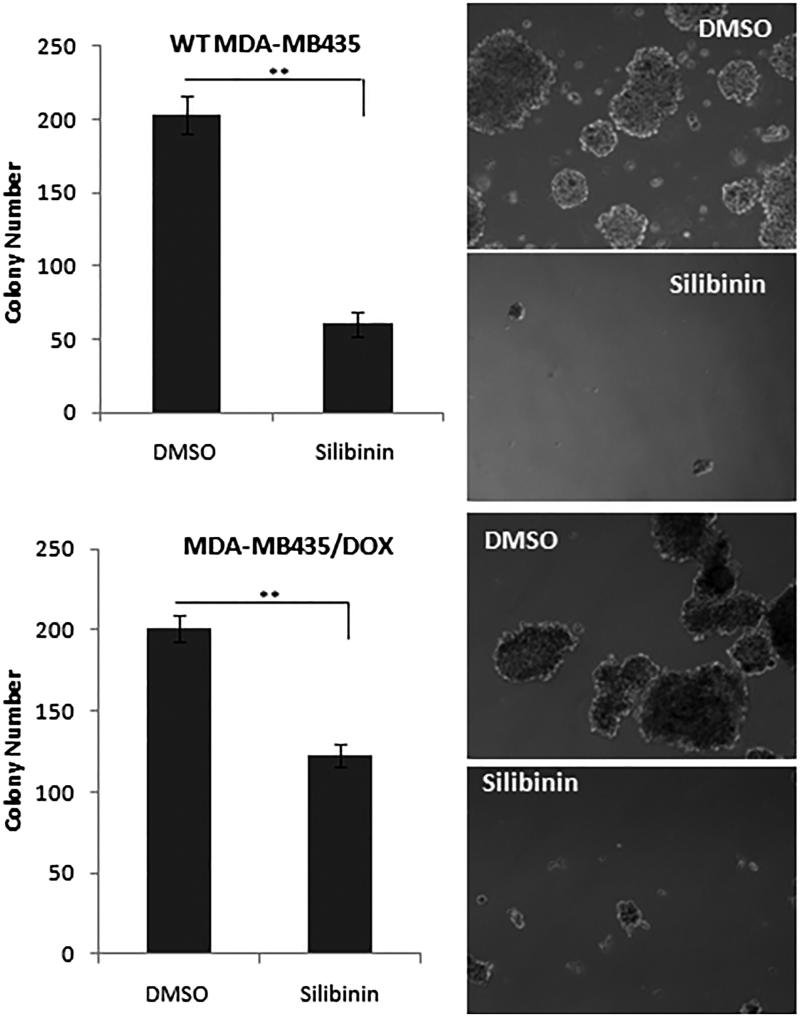
The effects of silibinin on tumorigenicity of MDA-MB-435. MDA-MB435/WT and MDA-MB435/DOX cells were treated with silibinin for 48 h then they are detached and subjected to mammosphere assay. The colonies were counted after 7 days. The right panel illustrates the morphology of the colonies (40× magnification).

Previous studies have shown that 3D spheres culture of tumour cells from various cancer cell lines including MDA-MB 435 have enriched cancer stem cell population with tumorigenic potential (Kondo 2007; Jang et al. 2015). To test the effects of silibinin on tumorigenic cancer stem like cell subpopulation of MDA-MB-435/WT and MDA-MB-435/DOX cells, the cells were treated with silibinin at the concentration of 250 and 400 μM, respectively. After 48 h of incubation, the cells were detached by trypsin then subjected to mammosphere culture system. As it is shown in Figure 5, silibinin significantly suppressed the formation of mammospheres in both MDA-MB435/WT and MDA-MB435/DOX cells. The number of colonies in MDA-MB-435/WT decreased from 215 in control group to 63 in the cell treated with silibinin. In MDA-MB435/DOX cells treated with silibinin, we observed a reduction of 40% in the number of colonies as compared with DMSO-treated cells.

### Silibinin suppresses the growth of MCF-7/PAC cells and sensitizes them to the cytotoxic effects of paclitaxel

**Figure 6.**
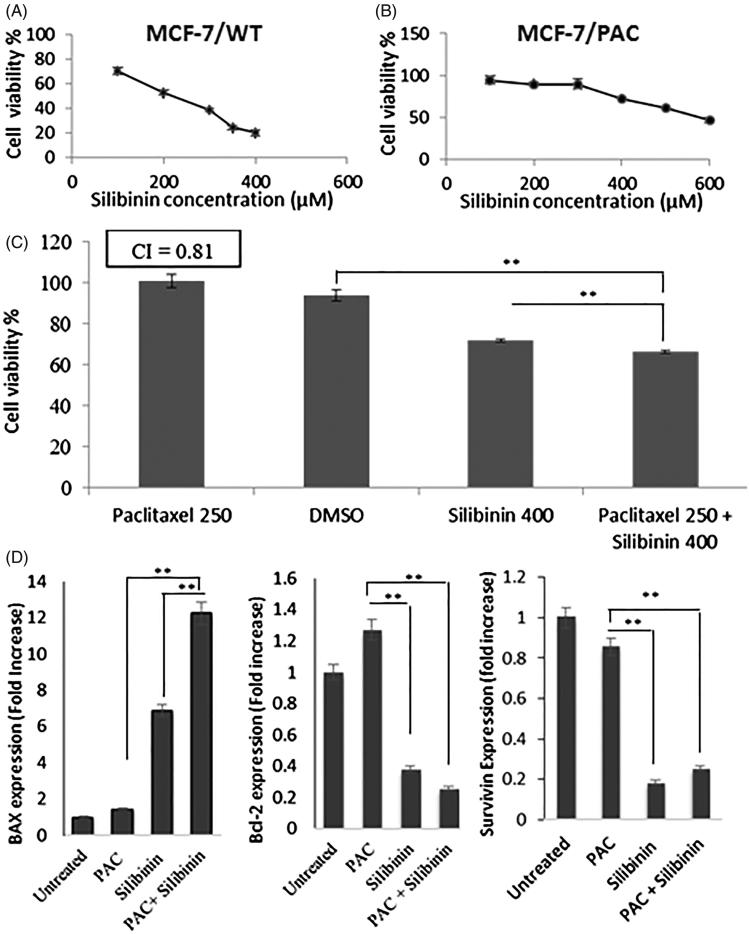
Growth inhibitory and chemo-sensitizing effects of silibinin in MCF-7 cells. Dose-response curve for the growth inhibitory effects of silibinin in (A) MCF-7/WT cell line and (B) MCF-7/PAC cell line. (C) The anticancer effects of PAC in combination with silibinin in MCF-7/PAC. (D) The effects of PAC and/or silibinin after 48 h incubation with MCF-7/PAC cells on the expression level of apoptosis-related proteins measured with RT-PCR.

To further confirm the anticancer and chemo-sensitizing effects of silibinin in breast cancer, we assessed the effects of this compound in the wild and drug-resistant phenotypes of another breast cancer cell line, MCF-7. Silibinin inhibited the growth of MCF-7/WT with the IC_50_ of 217 μM (Figure 6(A)) and induced a synergistic anticancer effect when it was used in combination with PAC (data not shown). CI for the anticancer effects of silibinin and PAC combinational treatment was found to be 0.8. Next we evaluated the effects of silibinin on MCF-7/PAC cells which has been developed and reported previously (Sharifi et al. 2014). As shown in the Figure 6(B), treatment of MCF-7/PAC cells with increasing concentrations of silibinin resulted in a dose-dependent decrease in the percentage of cell viability. The IC_50_ value for growth inhibitory effects of silibinin in MCF-7/PAC cells was found to be 572.3 μM. To examine whether silibinin can increase the therapeutic efficacy of chemotherapy against MCF-7/PAC cell line, the cells were treated with increasing concentration of PAC alone or in combination with silibinin at 400 μM concentration. As it has been illustrated in Figure 6(C), while treatment of MCF-7/PAC with PAC at the concentration of 250 nM did not result in significant cytotoxic effects in these cells (indicated by 100% of cell viability); treatment of these cells with PAC at the same concentration (250 nM) in combination with silibinin at 400 μM concentration resulted in a significant decrease (35% reduction) in the viability of these cells. The viability of the cells treated with silibinin at 400 μM concentration was 72% which was significantly higher than cell viability in the cells treated with the combination of these two compounds (65%). The CI for growth inhibitory effects induced by PAC in combination with silibinin was found to be 0.81 indicating a moderate synergistic anticancer effect of PAC and silibinin in MCF-7/PAC cells.

We have previously reported that the ratio of anti-apoptotic/pro-apoptotic proteins increases in MCF-7/PAC cells as compared to wild type MCF-7 cells suggesting that modulation in the expression level of apoptosis-associated proteins might be one of the important mechanisms of resistance development to PAC (Sharifi et al. 2014). Therefore we assessed the expression level of two important anti-apoptotic proteins (Bcl2, survivin) and BAX (a pro-apoptotic protein) in MCF7/PAC cells treated with PAC and/or silibinin. As it has been illustrated in Figure 6(D), treatment of MCF-7/PAC cells with PAC (250 nM) in combination with silibinin (400 μM) resulted in a 12-fold increase in the expression level of BAX as compared with control group. In the other hand, there was only a 2- and 7-fold increase in the expression level of BAX in MCF-7/PAC cells treated with either one of the compounds, PAC and silibinin, respectively. We also found a 75% decrease in the expression level of anti-apoptotic Bcl2 protein in MCF-7/PAC cell treated with combination of PAC and silibinin as compared with the control group. While we did not find any statistically significant effects on the expression level of Bcl2 in MCF-7/PAC treated with PAC, silibinin treatment resulted in a reduction of 60% in the level of Bcl2 (Figure 6(D)). Also we observed about an 80% decrease in the expression level of survivin in MCF-7/PAC cells treated with silibinin alone or in combination with PAC. Similar to Bcl2, we did not observe a significant decrease in the level of survivin in the cells treated with PAC alone as compared with control group (*p* > 0.05).

## Discussion

In this study, we have demonstrated that silibinin suppresses the growth of drug-resistant breast cancer cell lines and sensitize them to the cytotoxic effects of chemotherapy. The suppressive effects of silibinin on some of the key oncogenic proteins (i.e., STAT3 and Bcl2) correlates with the growth inhibitory and chemo-sensitizing effects of silibinin in drug-resistant breast cell lines. Our findings are in concordance with the previous studies showing that silibinin sensitizes cancer cells to anticancer drugs and provide compelling evidence for the therapeutic potential of this natural compound in treating drug-resistant cancers.

Consistent with the previous studies showing the anticancer effects of silibinin (Kil et al. 2014), we found that this compound significantly inhibits the growth of both wild-type and drug-resistant breast cell lines. Our observation on the synergistic effects of silibinin and chemotherapeutic agents (i.e., DOX and PAC) in breast cancer cell lines are in line with previous studies showing that silibinin enhances the killing effects of chemotherapeutic agents in various types of cancer models (Raina & Agarwal 2007). We observed a better synergistic anticancer effects generated by silibinin and DOX in MDA-MB435/WT cells as compared with what we found for combination of silibinin and PAC in MCF-7/WT (CI: 0.68 vs. 0.8). The reason behind this observation might be related to the suppressive effects of silibinin on the oncogenic STAT3 pathway which is constitutively active in MDA-MB435 breast cancer cells but not in MCF-7 cells (Bosch-Barrera & Menendez 2015). In support of this hypothesis we found that silibinin inhibits STAT3 activity in MDA-MB435 cells in a dose-dependent manner (Figure 4).

We also observed that silibinin significantly suppresses the activity of ERK and AKT pathways, both of which promote MDR and cancer progression in different types of human malignancies including breast cancer (Dhillon et al. 2007; Mundi et al. 2016; Navolanic et al. 2003; Steelman et al. 2011). AKT pathway is commonly found deregulated in wide range of cancers and plays a central role in cancer cells proliferation, angiogenesis, motility and survival (Mundi et al. 2016). The ERK pathway is well known for regulating cell proliferation and its constitutive activation has been observed in approximately, one-third of all human cancers. The significance of deregulated ERK pathway in tumorigenesis and acquisition of drug resistance in cancer is well documented by a large number of previously published studies (Dhillon et al. 2007). The suppression of AKT and ERK pathways by silibinin suggests another possible mechanism by which silibinin enhances the killing effects of DOX in MDA-MB435 cells.

Chemo-resistance is a major problem with the application of chemotherapeutic drugs in the treatment of breast cancer. While several studies have shown that silibinin enhances the cytotoxic effects of chemotherapeutic agents in cancer cells, the chemo-sensitizing effects of silibinin in drug-resistant cells have not been fully investigated (Raina & Agarwal 2007; Tyagi et al. 2004). Here we report that silibinin sensitizes two drug-resistant breast cell lines to chemotherapy and induce synergistic growth inhibitory effects when it is used in combination with chemo drugs in both cell lines. Although MDA-MB435/DOX and MCF7/PAC are two genotypically different cell lines, they share some similar mechanisms of drug resistance. Previous studies have shown that the increased levels of Bcl2 play an important role in the resistance of both MDA-MB435 and MCF7 cells to chemotherapy-induced apoptosis (Orlandi et al. 1999; Sharifi et al. 2014). The suppressive effects of silibinin on Bcl2 (Kauntz et al. 2012) can explain why the combination of silibinin and chemo drugs exerts synergistic anticancer effects in both of these two breast cancer cells lines.

In this paper, we have also studied the chemo-sensitizing effects of silibinin in a drug-resistant cell line (i.e., MDA-MB435/DOX) carrying constitutively active STAT3 which is shown to be important in MDR (Tan et al. 2014). We observed a 7-fold reduction in the IC_50_ of DOX in the cells treated with combination of DOX and silibinin as compared with the group treated with DOX only (10 vs. 71 μg/mL). Correlating with this observation, we found that silibinin suppresses STAT3 pathway (Figure 4) which has been shown to play a key role in cancer cell growth and resistance to chemotherapy (Al Zaid Siddiquee & Turkson 2008; Tan et al. 2014). Similar to our observation in wild type breast cells lines, the synergistic effects of silibinin and DOX in MDA-MB435/DOX was significantly greater than what we observed in MCF-7/PAC cells. We think the superior synergistic effects generated by silibinin and DOX in MDA-MB435/DOX cells might have resulted from the suppressive effects of silibinin on STAT3 pathway shown to be important for their growth and resistance to chemotherapy. Indeed constitutive activation of STAT3 has been reported to be one of the important mechanisms by which MDA-MB435/DOX acquire resistance to DOX (Ladas et al. 2010; Turkson et al. 2004). In the other hand, STAT3 is not active in MCF-7 cells and based on previous reports, an important mechanism of resistance to PAC is through changes in the level of anti-apoptotic (i.e., Bcl2) and pro-apoptotic proteins (i.e., BAX) (Sharifi et al. 2014).

In this study the growth inhibitory effect of PAC in combination with silibinin in MCF-7/PAC cells was found to be a moderate synergistic effect (CI = 0.8) which correlates with a significant increase in the expression level of BAX and a significance decrease in the level of Bcl2. Our findings on the effects of silibinin on apoptosis associated proteins are in line with other studies showing that modulation of anti-apoptotic and pro-apoptotic proteins is an important mechanism by which silibinin induce apoptosis in various types of cancers (Kauntz et al. 2012). Consistent with our observation on the synergistic effects of silibinin and PAC, Zhou et al. (2008) previously reported that silibinin can overcome resistance to paclitaxel in human ovarian cancer and sensitize PAC-resistant ovarian cancer cells to the cytotoxic effects of this chemotherapeutic agent.

One of the major reasons for us to study silibinin is related to the fact that it is known to carry a low toxicity in humans. For instance, in two clinical trials, silibinin phytosome (a commercial silibinin formulation) was administered orally to patients with prostate cancer at 13 g daily for a mean of 20 days or 2.5–20 g/daily for 28 days, respectively. This study reports no serious/acute toxicity in these patients receiving silibinin at the given does (Flaig et al. 2010). Furthermore, the lethal intravenous doses at 50% were reported to be 400 mg/kg in mice, 385 mg/kg in rats and 140 mg/kg in rabbits and dogs. We have also identified a number of other publications reporting the safe administration of relatively high doses of silibinin in patients (Cheung et al. 2010; Flaig et al. 2007; Hoh et al. 2006; Mulrow et al. 2000; Ting et al. 2013; Zhang et al. 2012). In addition to the excellent safety profile of silibinin, this compound is a hepatoprotective agent and there are several case reports suggesting a beneficial role of silibinin for the treatment of chemotherapy-induced hepatotoxicity (Comelli et al. 2007; Ladas et al. 2010).

Multiple studies have shown that silibinin potently inhibits metastasis, pinpointing to its potential for treatment of metastatic malignancies (Singh et al. 2008; Wu et al. 2013). In this regard, breast cancer has a metastatic tendency, the majority of patients with this type of malignancy are diagnosed at advanced III and IV stage disease, and these patients often display systemic diseases with a high propensity for dissemination to extra nodal sites such as liver, bone and lung (Scully et al. 2012). Taken together, the suppressive effects of silibinin on tumor metastasis, as well as its sensitizing effects on drug-resistant cells, argue that silibinin is a promising drug to treat breast cancer. The major limitation to the clinical use of silibinin in cancer is its poor pharmacokinetics properties. Research projects are currently underway in our lab to increase the water solubility of silibinin and improve its pharmacokinetic properties by encapsulation of this drug in nanoparticles which could be used systemically. Nanoparticles of silibinin are expected to accumulate in tumour tissue and deliver a high concentration of drug to cancer cells.

## Conclusions

In conclusion, our findings revealed that silibinin suppresses the growth of both wild type and drug-resistant MDA-MB-435 and MCF-7 breast cell lines. We also found that silibinin significantly increases the cytotoxic effects of DOX and PAC in the breast cancer cell lines resistant to these chemotherapeutic agents. The anticancer effects induced by silibinin in combination with DOX or PAC in both wild-type and drug-resistant breast cancer cells were found to be synergistic effects. Correlating with these observations, we found a significant decrease in the level of key oncogenic proteins including STAT3, ERK, and survivin in MDA-MB435 cells. The synergistic effect of silibinin and PAC in MCF-7/PAC cells was correlated with a marked increase in the level of BAX (a pro-apoptotic protein) and a significant decrease in the level of two important anti-apoptotic proteins, Bcl2 and survivin. All together, these findings suggest that silibinin might be a useful drug for treatment of breast cancers.

## Supplementary Material

Ommoleila_Molavi_et_al_supplemental_content.zip
