# Supplementary material for: Silibinin sensitizes chemo-resistant breast cancer cells to chemotherapy
Source: Pharm Biol. 2016 Dec 27;55(1):729–39. doi: 10.1080/13880209.2016.1270972 (PMC6130726; doi:10.1080/13880209.2016.1270972)
Supplement: Ommoleila_Molavi_et_al_supplemental_content.zip [file IPHB_A_1270972_SM8206.zip › Ommoleila Molavi et al supplemental content.pdf]

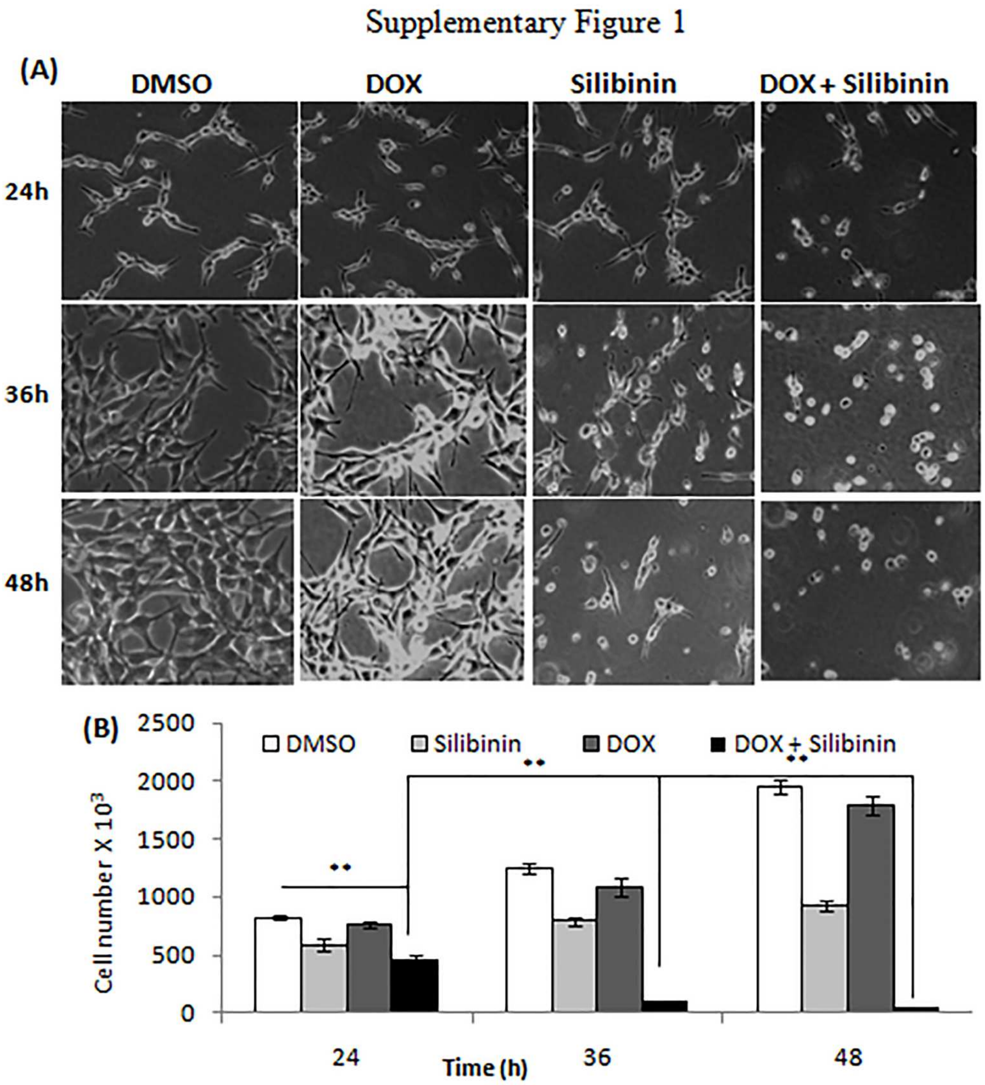

Supplementary Figure 1: Growth inhibitory effects of silibinin and DOX combination therapy in MDA-MB 435/WT cell line. (A) Morphological evaluation of MDA-MB-435/WT cells receiving different treatments at various time points (40x magnification). (B) Trypan blue assay of the cells in all four the experimental groups at the selected time points.

168x186mm (300 x 300 DPI)
